# Complete Genome Sequence of *Rice Tungro Bacilliform Virus* Infecting Asian Rice (Oryza sativa) in Malaysia

**DOI:** 10.1128/MRA.00262-19

**Published:** 2019-05-16

**Authors:** Maathavi Kannan, Maisarah Mohamad Saad, Noraini Talip, Syarul Nataqain Baharum, Hamidun Bunawan

**Affiliations:** a Institute of Systems Biology, Universiti Kebangsaan Malaysia, Bangi, Malaysia; b Rice Research Centre, MARDI Seberang Perai, Kepala Batas, Penang, Malaysia; c School of Environmental and Natural Resource Sciences, Faculty of Science and Technology, Universiti Kebangsaan Malaysia, Bangi, Malaysia; DOE Joint Genome Institute

## Abstract

Rice tungro disease was discovered in Malaysia in the 1930s. The first and only genome of *Rice tungro bacilliform virus* (RTBV) isolated from rice in Malaysia was sequenced in 1999. After nearly two decades, here, we present the complete genome sequence of an RTBV isolate in rice from Seberang Perai, Malaysia.

## Abstract (precis)

Rice tungro disease was discovered in Malaysia in the 1930s. The first and only genome of *Rice tungro bacilliform virus* (RTBV) isolated from rice in Malaysia was sequenced in 1999.

## ANNOUNCEMENT

Rice tungro disease (RTD) is known to be one of the most economically important viral diseases of rice (1). High incidences of RTD have been reported across South and Southeast Asia (2, 3). Every year, RTD causes losses of approximately $1.5 billion in rice yield (4). The disease results from an infection by two distinct viruses, *Rice tungro bacilliform virus* (RTBV) and *Rice tungro spherical virus* (RTSV) (5, 6). The severity of tungro symptoms is driven by RTBV (7), while RTSV mainly plays a role as a helper virus in vector transmission of RTBV (8).

RTBV is a member of the genus Tungrovirus and family *Caulimoviridae* (9). RTBV has a circular double-stranded DNA genome of about 8 kb (10). The genome of RTBV (Philippines isolate) was sequenced for the first time in 1991 (11), followed by complete sequencing of five biological variants, Phi-2 (12), Phi-3 (13), Ic, G1, and G2 (14). Since then, several Indian isolates from Chinsura (15), Kanyakumari (2), Andra Pradesh, and West Bengal (16) were sequenced. Moreover, complete genomes of isolates from Punjab (17), Chainat, and Serdang (18) have also been reported.

Despite the importance of tungro viruses, less research work has been conducted on RTBV in Malaysia (19). The only genome of RTBV in Malaysia, RTBV-Serdang (GenBank accession no. AF076470), was sequenced in 1999 (18). Since then, no RTBV genome sequence has been reported in the past 19 years, although the disease is still endemic in Malaysia (9). In this study, we report the complete genomic sequence of an RTBV isolate obtained from an infected field. This will enable further studies regarding RTBV evolution and genome variability in Malaysia.

Rice plants exhibiting RTD symptoms were collected from a paddy field in Seberang Perai, Malaysia. The cetyltrimethylammonium bromide (CTAB) method was utilized in the extraction of DNA from infected leaves of rice plants (20). Designation of five overlapping primer pairs was done (Table 1) based on the aligned complete genome sequences of 13 RTBV isolates derived from GenBank (https://www.ncbi.nlm.nih.gov/nuccore/?term=rice+tungro+bacilliform+virus+complete+genome). PCR amplification of those five fragments covering the entire genome from the RTBV DNA template was carried out using PCRBIO HiFi polymerase, and the products were electrophoresed on a 1% agarose gel. Once the band size was confirmed, the products were purified from the gel using a QIAquick gel extraction kit (Qiagen, Malaysia) and cloned into pJET1.2 vector. A minimum of two recombinant plasmids for every fragment were sent to the First BASE Laboratories Sdn Bhd company for sequencing in forward and reverse directions using the Sanger sequencing method.

**TABLE 1:** Primers used in PCR to amplify the complete genome of *Rice tungro bacilliform virus*

Primer name	Primer sequence	Product size (nucleotides)	Primer position^*a*^
RTBVF1	TGGTATCAGAGCGATGTTCG	1,357	1–20
RTBVR1	ATGGCCATCATGCCTATATG		1333–1352
RTBVF2	CATATAGGCATGATGGCCAT	1,386	1333–1352
RTBVR2	GTCCATCCAAGACCACAT		2695–2712
RTBVF3	ATGTGGTCTTGGATGGA	1,158	2695–2711
RTBVR3	TGCTCTCATAGCTAATG		3836–3852
RTBVF4	CATTAGCTATGAGAGCA	1,972	3836–3852
RTBVR4	GATATGCTCAAAGGTAGGCT		5771–5790
RTBVF5	AGCCTACCTTTGAGCATATC	2,264	5771–5790
RTBVR5	TTTCTAGGCACCCCCCT		8000–8016

a Primer positions are based on the Serdang isolate (GenBank accession no. AF076470).

A total of 10 bidirectional reads were obtained. The program Clustal Omega version 1.2.4 with default parameters was used to align all obtained reads in pairs to generate respective consensus nucleotide sequences of five fragments (21). Adjacent segments with overlapping sequences were joined in order to generate the complete genome of RTBV.

The full-length genome sequence of RTBV isolated from Seberang Perai (RTBV-SP) was thus obtained and found to be 8,000 nucleotides in length, with a G+C content of 33.3%. Searches through BLASTN revealed that the nucleotide sequence of the RTBV-SP isolate is 81.45% to 95.44% identical to those of other RTBV complete genomes available in the GenBank database. The highest nucleotide similarity (95.44%) was observed with the Serdang isolate. Interestingly, the RTBV-SP genome is shorter than that of the Serdang isolate by 16 nucleotides.

This study revealed that the genetic makeup of RTBV-SP has remained stable despite a time gap of approximately 20 years between genome sequencing of the SP isolate and the Serdang isolate. Availability of the data on RTBV variability in Malaysia would be helpful in determining the resistance strategies against tungro.

### Data availability.

The complete genomic sequence of the RTBV-SP isolate was submitted to NCBI GenBank with the accession no. MK552377.
